# Supplementary material for: High-density frustrated Lewis pairs based on Lamellar Nb2O5 for photocatalytic non-oxidative methane coupling
Source: Nat Commun. 2023 Apr 10;14:2000. doi: 10.1038/s41467-023-37663-x (PMC10086065; doi:10.1038/s41467-023-37663-x)
Supplement: Supplementary file 1 — Supplementary Information [file 41467_2023_37663_MOESM1_ESM.pdf]

Supporting Information

High-density Frustrated Lewis Pair Based on Lamellar Nb_2O_5 for Photocatalytic Non-Oxidative Methane Coupling

Ziyu Chen[1], Yutao Ye[1], Xiaoyi Feng[1], Yan Wang[1], Xiaowei Han[1], Yu Zhu[1], Shiqun Wu[1], Senyao Wang[1], Wenda Yang[1], Lingzhi Wang[1*], Jinlong Zhang[1*]

[1]Shanghai Engineering Research Center for Multi-Media Environmental Catalysis and Resource Utilization, Key Lab for Advanced Materials and Joint International Research Laboratory of Precision Chemistry and Molecular Engineering, Feringa Nobel Prize Scientist Joint Research Center, Institute of Fine Chemicals, School of Chemistry and Molecular Engineering, East China University of Science & Technology, 130 Meilong Road, Shanghai, 200237, China. E-mail: wlz@ecust.edu.cn; jlzhang@ecust.edu.cn.

Supplementary Table 1 The structural parameters of the Nb_2O_5 obtained from XRD analysis.

Catalyst	peak	hkl	Crystalline size (nm)	Dislocation density δ *10^{-3} lines/m^2	Strain ε
Nb_2O_5	22.7	001	17.61	3.22	0.010
H_2-Nb_2O_5	22.4	001	17.20	3.38	0.010
673K-Nb_2O_5	22.7	001	14.09	5.04	0.012
	32.3	250	13.05	5.87	0.009
773K-Nb_2O_5	22.4	001	14.07	5.09	0.013
	32.1	250	13.98	5.12	0.009
873K-Nb_2O_5	22.6	001	13.43	5.54	0.013
	32.3	250	12.77	6.13	0.010
973K-Nb_2O_5	22.4	001	15.38	4.23	0.011
	32.1	250	16.97	3.47	0.007

Supplementary Table 2 The cell parameters of the Nb_2O_5 obtained from (001) peak analysis.

Catalyst	a	b	c	α	β	γ
Nb_2O_5	6.15	29.15	3.94	90	90	90
H_2-Nb_2O_5	6.15	29.13	3.93	90	90	90
673K- Nb_2O_5	6.14	29.15	3.93	90	90	90
773K- Nb_2O_5	6.17	28.94	3.95	90	90	90
873K- Nb_2O_5	6.95	28.00	3.96	90	90	90
973K- Nb_2O_5	6.12	28.77	3.95	90	90	90

Supplementary Table 3 Results of various samples for photocatalytic NOCM[a]

Rate(μmol/g h)	H_2	Er±	C_2H_6	Er±	C_3H_8	Er±	C_4H_{10}	Er±	CH_4 conversion	Er±	Ethane selectivity
Nb_2O_5	29.7	4.1	32.3	3.5	0.7	0.5	0.0	0.0	66.8	5.8	97.9%
H_2-Nb_2O_5	249.1	7.4	217.2	14.2	13.0	1.0	2.6	0.6	484.1	32.8	93.3%
673K-Nb_2O_5	319.3	8.6	284.2	9.4	12.4	1.5	2.2	0.3	614.5	21.9	95.1%
773K-Nb_2O_5	351.3	13.5	353.9	13.3	15.8	1.7	3.2	1.5	767.8	24.4	94.9%
873K-Nb_2O_5	780.9	8.6	600.8	4.3	62.3	1.1	17.0	1.1	1456.5	11.2	88.3%
973K-Nb_2O_5	567.4	5.3	535.5	6.5	25.6	1.3	5.6	1.1	1170.1	5.4	94.5%

[a] Reaction conditions were as follows: samples, 0.005 g; reaction time, 4 h; reactant, 45 mL of methane; light source, 300 W Xe lamp; reaction temperature, room temperature; quartz reactor, 45 cm^3. The products were analyzed by gas chromatography with flame-ionization and a thermal conductivity detector.

Supplementary Table 4 Representative works on photocatalytic gas phase methane reaction.

Samples	Reactions	Conditions	Methane conversion to expected product (μmol g^{-1} $h^{-1)}$	References
Au/m-ZnO	NOCM[a]	0.001 g sample, 300 W Xe lamp, Ar (0.1 MPa) CH_4 (22.3 mL)	26.6	2018[1]
Ru/Zn-g-C_3N_4	POM[b]	0.1 g sample, 150 W Xe lamp, 0.5 bar mixed gas (CO_2/CH_4/Ar=7.5:7.5:85)	1360.3	2019[2]
Zn-HPW/TiO_2	POM	0.1 g sample, 400W Xe lamp for 6 h, 200 μmol CH_4, 0.3 MPa, Air 0.1 MPa.	429	2019[3]
Pt/HGTS (2%)	NOCM	0.2 g sample, 40 W Xe lamp for 4 h; 44.6 μmol CH_4.	3.48	2019[4]
Ti_3AlC_2/TiO_2	DRM[c]	0.15 g sample,150 mW cm^{-2} UV light, mixed gas (CO_2:CH_4=1:1)	909	2020[5]
Au/TiO_2	NOCM	0.005 g sample,100 mW cm^{-2} AM 1.5G, CH_4/Ar=1:9,GHSV=120 000 g^{-1} h^{-1}	163.4	2020[6]
Ag-HPW/TiO_2	NOCM	0.1 g sample, UV light for 7 h, CH_4 0.3 MPa	46	2020[7]
2Nb-TS	NOCM	0.1 g sample, 40 W Xe lamp for 4 h; 44.6 μmol CH_4.	3.57	2021[8]
ZnO-AuPd	NOCM	0.002 g sample, 300 W Xe lamp for 4 h, 22.3 μmol CH_4.	134	2021[9]
Pt/Ga_2O_3	NOCM	0.2 g sample,300 W Xe lamp for 4 h, 44.6 μmol CH_4.	134	2021[10]
GaN:ZnO solid solutions	NOCM	0.002 g sample,200 nm-400 nm light for 2 h， 300 μmol CH_4.	331	2021[11]
$Cu_{0.1}Pt_{0.5}$/PC-50	POM	0.1 g sample, 40 W LED lamp(λ=365 nm), O_2/CH_4=1:40 GHSV=24000 g^{-1} h^{-1}	1456.8	2022[12]
873K-Nb_2O_5	NOCM	0.005 g sample, 300 W Xe lamp for 4 h; 44.6 mL CH_4.	1456	This work

[a] Non-oxidative Coupling of Methane (NOCM), the reactant is only methane, excluding any oxidant, and the products are multi-carbon alkanes and alkenes. [b] Photocatalytic Partial Oxidation of Methane (POM), reactants contain methane and oxidizing gases, and products consisting of high-value-added C_2 hydrocarbons and C_1 platform molecules such as CO, CH_3OH, and HCHO. [c] Photocatalytic Dry Reforming of Methane (DRM), the oxidizing gas is usually CO_2, and the product is usually mainly syngas.

Supplementary Table 5 Results of various samples for long-time photocatalytic NOCM.

Yield	Nb_2O_5				H_2-Nb_2O_5				873K-Nb_2O_5				
(μmol/g)	C_2H_6	C_3H_8	C_4H_{10}	CH_4 conversion	C_2H_6	C_3H_8	C_4H_{10}	CH_4 conversion	C_2H_6	C_3H_8	C_4H_{10}	C_5H_{12}	CH_4 conversion
4h	131	1.87	0	269	803	49	8	1786	2363	258	85	0	5846
8h	168	2.51	0	344	1338	68	8	2913	4374	332	84.6	4.48	10107
12h	186	4.2	1.2	389	1854	82.2	15	4014	5732	318	99	21	12923
16h	190	4.5	1.6	399	2027	105	23	4461	6600	454	102	59.2	15265
20h	193	5.1	2.4	410	2190	137.8	23.9	4889	7030	653	128	114	17100

Supplementary Table 6 Results of 873K-Nb_2O_5 sample for cycle NOCM[a].

Rate (μmol/g h)	H_2	Er±	C_2H_6	Er±	C_3H_8	Er±	C_4H_{10}	Er±	CH_4 conversion	Er±
First	780.9	8.6	600.8	4.3	62.3	1.1	17.0	1.1	1456.5	11.2
Second	752.3	3.4	587.7	5.4	66.3	3.4	18.0	4.4	1446.4	18.6
Third	704.9	14.8	625.0	14.9	28.0	2.3	12.9	2.8	1385.7	17.0
Forth	580.2	7.5	550.5	9.9	16.2	2.8	11.4	1.2	1186.9	30.6

[a]Cycle reaction conditions were as follows: after the photocatalytic NOCM reaction, the catalyst was vacuumed for 4h to remove adsorbed gas and water molecules from the air.

Supplementary Table 7 Average activity of various samples in flow-type reactor photocatalytic NOCM.

Samples	Ethane (μmol min^{-1})	Hydrogen (μmol min^{-1})	Selectivity（C_2H_6/H_6）
Nb_2O_5	-	-	-
H_2-Nb_2O_5	-	-	-
673K-Nb_2O_5	-	-	-
773K-Nb_2O_5	0.106	0.105	1.01
873K-Nb_2O_5	0.39	0.40	0.97
973K-Nb_2O_5	0.14	0.14	1

Supplementary Table 8. Results of AQY for methane conversion at different wavelengths.

Wavelength (nm)	Produced hydrogen (μmol)	Light power density I (mW cm^{-2})	AQY (%)
365	58.8	22	0.43
420	27.4	115	0.033
475	1.68	212	0.00042

Supplementary Note 1:

The AQY measurement experiment was carried out in a quartz reactor with an illumination diameter of 6 cm, the reaction time was 4 hours. In order to ensure that the catalyst covers the illuminated area, the catalyst was 0.5 g.

The Apparent quantum yield (AQY) can be calculated as AQY=100%× $N_{reacted}$ / $N_{incident}$.

We use produced hydrogen to calculate the reacted electrons. The number of the reacted electrons is: $N_{reacted}$=2×the number of the produced hydrogen molecules.

The number of the incident photons is: $N_{incident} = \frac{pt}{hv} = \frac{pt\lambda}{hc}$. In the equation, t is the time (s), h is Planck's constant, v is the light frequency, and c is the speed of light in free space.

The light power is: P=IS. The projected area of the reactor was circular with a diameter of 6.0 cm. therefore, the irradiated area (S) is: S=π×3.0×3.0=28.27 cm^2.

Take the estimation of the AQY at 365 nm as an example:

$N_{incident}=22\times28.27\times10^{-3}\times4\times60\times60\times365\times10^{-9}\div(3\times10^{8}\times6.626\times10^{-34})\approx1.64\times10^{22}$

$AQY=100\%\times2\times58.8\times10^{-6}\times6.02\times10^{23}\div(1.64\times10^{22}) \approx 0.43\%$

Supplementary Table 9. Residual coke after NOCM reaction.

Samples	Weight (mg)	C Area	C (%)
Nb_2O_5 (20h)	2.2440	20908	30.79
H_2-Nb_2O_5 (20h)	1.7320	21484	40.99
873K-Nb_2O_5 (4h)	2.1090	340	0.6
873K-Nb_2O_5 (12h)	2.2040	421	0.69
873K-Nb_2O_5 (20h)	1.8530	384	0.76

Supplementary Table 10. Radiative fluorescence lifetimes of the photo-induced charge carriers.

Catalyst	τ_1 (ns)	A_1	τ_2 (ns)	A_2	τ^a (ns)
Nb_2O_5	0.0663	100%	-	-	0.0663
673K-Nb_2O_5	0.0824	100%	-	-	0.0824
773K-Nb_2O_5	0.0575	99.6%	6.0003	0.4%	1.8178
873K-Nb_2O_5	0.0586	99.69%	9.8990	0.31%	3.4475
973K-Nb_2O_5	0.0634	99.49%	5.3154	0.51%	1.6421

[a] The average lifetime τ for the catalysts is calculated by the formula $\tau = (A_1\tau_1^2 + A_2\tau_2^2 + A_3\tau_3^2) / (A_1\tau_1 + A_2\tau_2 + A_3\tau_3)$.

Supplementary Table 11. Results of photocatalytic NOCM after the acidic and basic sites quenching [a]

Yield	873K-Nb_2O_5				H_2-Nb_2O_5				Nb_2O_5		
(μmol/g h)	C_2H_6	C_3H_8	C_4H_{10}	CH_4 conversion	C_2H_6	C_3H_8	C_4H_{10}	CH_4 conversion	C_2H_6	C_3H_8	CH_4 conversion
original	601	62	17	1456	217	13	2.6	484	-	-	-
Pyridine adsorption	312	17	0	675	18.5	0.6	0	38.8	-	-	-
Pyrrole adsorption	98	0	0	196	8.5	0	0	17	-	-	-

[a] 0.005 g sample was quenched by 0.5 mL pyridine and pyrrole for 0.5 h, respectively; Adsorbed liquid was removed by the vacuuming reaction.

Supplementary Table 12. Results of methane adsorption capacity of different samples.

Catalyst	mmol/g
Nb_2O_5	0.066
H_2-Nb_2O_5	0.138
873K-Nb_2O_5 after pyrrole adsorption	0.192
873K-Nb_2O_5 after pyridine adsorption	0.214
873K-Nb_2O_5	0.488

Supplementary Table 13. Curvefit parameters[a] of Nb K-edge EXAFS for Nb_2O_5 samples

samples	Path	Coordination number[b]	Radial distance[c]/ Å	$\sigma^{2[d]}$/ Å^2	ΔE_0	*R*-factor
Nb_2O_5	Nb-O	6.35(0.30)	2.13(0.02)	0.005(±0.002)	9.37(1.09)	0.007
H_2-Nb_2O_5	Nb-O	5.63(0.41)	2.12(0.10)	0.005(±0.002)	8.97(1.33)	0.014
673K-Nb_2O_5	Nb-O	4.64(0.67)	2.09(0.06)	0.007(±0.003)	8.64(1.46)	0.014
		1.54(0.33)	2.22(0.06)	0.007(±0.003)	8.64(1.46)	
773K-Nb_2O_5	Nb-O	4.19(0.69)	2.08(0.06)	0.008(±0.005)	9.11(2.20)	0.014
		2.09(0.68)	2.21(0.06)	0.008(±0.005)	9.11(2.20)	
873K-Nb_2O_5	Nb-O	2.92(0.68)	2.01(0.01)	0.009(0.001)	6.23(2.15)	0.024
		2.81(0.83)	2.18(0.01)	0.009(0.001)	6.23(2.15)	
973K-Nb_2O_5	Nb-O	4.06(0.93)	2.02(0.01)	0.006(0.005)	6.93(0.92)	0.004
		2.01(0.37)	2.19(0.01)	0.006(0.005)	6.93(0.92)	

[a] S_0^2 was fixed as 0.9, ΔE_0 was refined as a global fit parameter, returning a value of (-3 ± 3) eV. Data ranges：2.0 ≤ k ≤ 11.0 Å^{-1}, 1.0 ≤ R ≤ 2.0 Å. The number of variable parameters is 4, out of a total of 7.3 independent data points. [b]These coordination numbers were constrained as N(Nb-O)=1 based on the different Nb-O paths from the different crystal structures. The distances for Nb-O paths are from the FEFF file of the standardized different Nb_2O_5 cell data. [c] The radial distances are not phase-corrected and do not represent actual interatomic distances. [d]The Debye-Waller factors were constrained as σ^2 of all the Nb-O paths are equal which is for decreasing the correlation.

Supplementary Note 2:

Considering asymmetry and distortion in the crystal structure of $NaBH_4$-treated Nb_2O_5, and the wide Nb-O distance distribution range, as few paths as possible were chosen to investigate the overall situation of the first coordination shell. For the cases of pristine and H_2-treated Nb_2O_5, representative fits can be achieved through one average Nb-O path. The complex situation of $NaBH_4$-treated Nb_2O_5 requires two paths. One belongs to the shorter Nb-O path in the lattice, and one Nb-O path is due to the joint contribution of the severe distortion of the lattice and the elongation of the surface Nb-OH. It is worth noting that the fitting results show a simplified average coordination environment.

Supplementary Table 14. Brønsted acid and Lewis Acid of $NaBH_4$-treated Nb_2O_5 Samples.

mmol/g	Brønsted acid	Lewis acid
673K-Nb_2O_5	0.01061	0.04194
773K-Nb_2O_5	0.01550	0.05895
873K-Nb_2O_5	0.02348	0.06039
973K-Nb_2O_5	0.02193	0.04851

Supplementary Table 15. The acid of Nb_2O_5 Samples analysis by NH_3-TPD.

Catalyst	Total acid site(mmol/g)	Strong site 1	Strong site2
Nb_2O_5	-	-	-
H_2-Nb_2O_5	0.030378	0.030378	-
673K-Nb_2O_5	0.091437	0.029944	0.061493
773K-Nb_2O_5	0.126565	0.04082	0.085745
873K-Nb_2O_5	0.150966	0.048765	0.102201
973K-Nb_2O_5	0.123974	0.03982	0.084154

Supplementary Table 16. The calculated distance (angstrom) and Mulliken Charge of $NaBH_4$-treated Nb_2O_5 models.

	dNb_{LA}-OH_{Lb} (Å)[a]	Mulliken Charge of Nb_{LA}	Mulliken Charge of Nb_{LB}	Mulliken Charge of –OH_{LB}
Vo^{4g}-Nb^1-O-Nb^2-OH	4.806	1.977	2.061	-0.304
Vo^{4g}-Nb^1-O-Nb^3-OH	4.556	1.972	2.035	-0.213
Vo^{4g}-Nb^2-O-Nb^1-OH	4.801	1.957	2.133	-0.248
Vo^{4g}-Nb^2-O-Nb^3-OH	4.271	1.955	2.024	-0.213
Vo^{4g}-Nb^3-O-Nb^1-OH	4.875	1.956	2.141	-0.253
Vo^{4g}-Nb^3-O-Nb^2-OH	3.485	1.950	2.003	-0.305

[a] dNb_{LA}-OH_{Lb} represents the distance between the exposed Nb site (LA site) and oxygen in –OH (LB site).

Supplementary Table 17. The calculated Mulliken Charge of H_2-treated Nb_2O_5 models.

	Mulliken Charge of Nb_{LA}
Vo^{4g}-Nb^1	1.956
Vo^{4g}-Nb^2	1.951
Vo^{4g}-Nb^3	1.950

Supplementary Table 18. The calculated Mulliken Charge of Nb-OH models before and after methane activation.

	Mulliken Charge of $CH_3^{\delta-}$	Mulliken Charge of Nb_{LB}	Mulliken Charge of –OH_{LB}
Nb^1-OH	-	2.096	-0.194
Nb^2-OH	-	2.031	-0.155
Nb^3-OH	-	2.022	-0.218
Nb^1-OH (after methane activation)	-0.240	2.065	-0.390
Nb^2-OH(after methane activation)	-0.217	1.976	-0.349
Nb^3-OH(after methane activation)	-0.263	1.972	-0.352

Supplementary Table 19. The calculated Mulliken Charge of $NaBH_4$-treated Nb_2O_5 models after methane activation.

	Mulliken Charge of $CH_3^{\delta-}$	Mulliken Charge of Nb_{LA}	Mulliken Charge of Nb_{LB}	Mulliken Charge of –OH_{LB}
Vo^{4g}-Nb^1-O-Nb^2-OH	-0.308	2.018	1.987	-0.374
Vo^{4g}-Nb^1-O-Nb^3-OH	-0.311	2.016	1.986	-0.395
Vo^{4g}-Nb^2-O-Nb^1-OH	-0.235	1.961	2.073	-0.384
Vo^{4g}-Nb^2-O-Nb^3-OH	-0.320	1.979	1.975	-0.364
Vo^{4g}-Nb^3-O-Nb^1-OH	-0.346	2.081	2.071	-0.376
Vo^{4g}-Nb^3-O-Nb^2-OH	-0.225	1.964	1.995	-0.394

Supplementary Table 20. The calculated distance (angstrom) and energy of transition state of C–H bond cleavage.

	dVo^{4g}-Nb^1…$CH_3^{\delta-}$(Å)[a]	dNb^3-OH…$H^{\delta+}$(Å)[b]	$dCH_3^{\delta-}$…$H^{\delta+}$ (Å)[c]	Energy (eV)
Vo^{4g}-Nb^1-O-Nb^3-OH(TS)	2.111	1.509	1.567	0.28
Vo^{4g}-Nb^1-O-Nb^3-OH	1.748	1.050	3.126	-1.13
Nb^3-OH(TS)	-	1.880	1.898	0.43
Nb^3-OH	-	1.039	3.496	0.054
Vo^{4g}-Nb^1(TS)	2.462	-	-	2.18
Vo^{4g}-Nb^1	-	-	-	0.21

[a]dVo^{4g}-Nb^1…$CH_3^{\delta-}$ represents the distance between Nb^1 and C atom. [b] dNb^3-OH…$H^{\delta+}$ represents the distance between O atom and H atom from methane. [c]d $CH_3^{\delta-}$…$H^{\delta+}$ represents the distance between C atom and H atom from the cleavage C-H bond.

Supplementary Table 21. The calculated bond lengths (angstrom) and angles (degree) of different models.

	dNb^1-Nb^3 (Å)[a]	dNb^1-Nb^2(Å)	dNb^2-Nb^3(Å)	dO-H(Å)	∠Nb^3-O-H(°)
cluster	3.980	4.108	3.973	--	--
Vo^{4g}-Nb^1	4.198	4.2	4.016	--	--
Nb^3-OH	4.180	4.186	4.024	0.986	123.153
Vo^{4g}-Nb^1-O-Nb^3-OH(GS)	4.178	4.192	4.024	0.989	121.922
Vo^{4g}-Nb^1-O-Nb^3-OH(ES)	4.178	4.191	4.024	0.989	121.920

[a]dA−B represents the bond lengths between A and B atoms and ∠Nb-O-H represents the Nb-O-H angle.

Supplementary Table 22. Brønsted acid and Lewis Acid of 873K-Nb_2O_5 before and after light irradiation.

mmol/g	Brønsted acid	Lewis acid
Dark	0.03113	0.05931
Light	0.03325	0.08004

Supplementary Table 23. The calculated adsorption energies of $CH^{3\delta-}$ and $H^{\delta+}$ on Vo^{4g}-Nb^1 and Nb^3-OH.

Adsorption Energy (eV)	Vo^{4g}-Nb^1⋯$CH_3^{\delta-}$	Vo^{4g}-Nb^1⋯$H^{\delta+}$	Nb^3-OH⋯$CH_3^{\delta-}$	Nb^3-OH⋯$H^{\delta+}$
Ground state	-1.13	-0.16	-0.29	-1.07
Excited state	-1.59	-0.18	-0.27	-1.34

Supplementary Table 24. Vibrational Frequencies of Free CH_4, Observed IR Bands upon CH_4 adsorption on pristine Nb_2O_5 and 873K-Nb_2O_5 (in cm^{-1})

Vibrational modes	Gas phase[a]	Adsorbed on pristine Nb_2O_5	Adsorbed on 873K-Nb_2O_5
v_1 , symmetric stretch.	2914[b]	Not observed	2822

[a]Reference [13] [b]The symmetric stretching mode v_1 is IR forbidden by symmetry and their frequencies were measured by means of Raman spectroscopy.

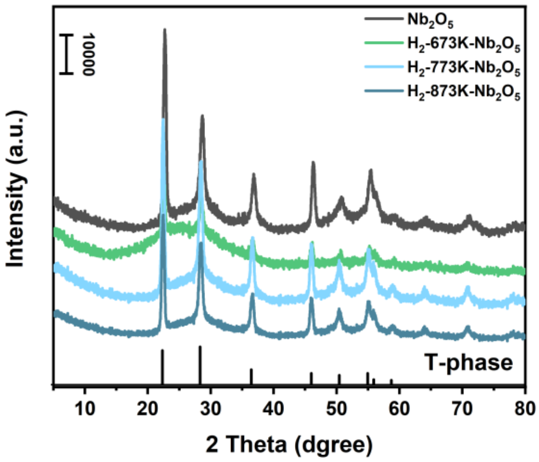

Supplementary Figure 1. XRD patterns of H_2-treated Nb_2O_5 samples.

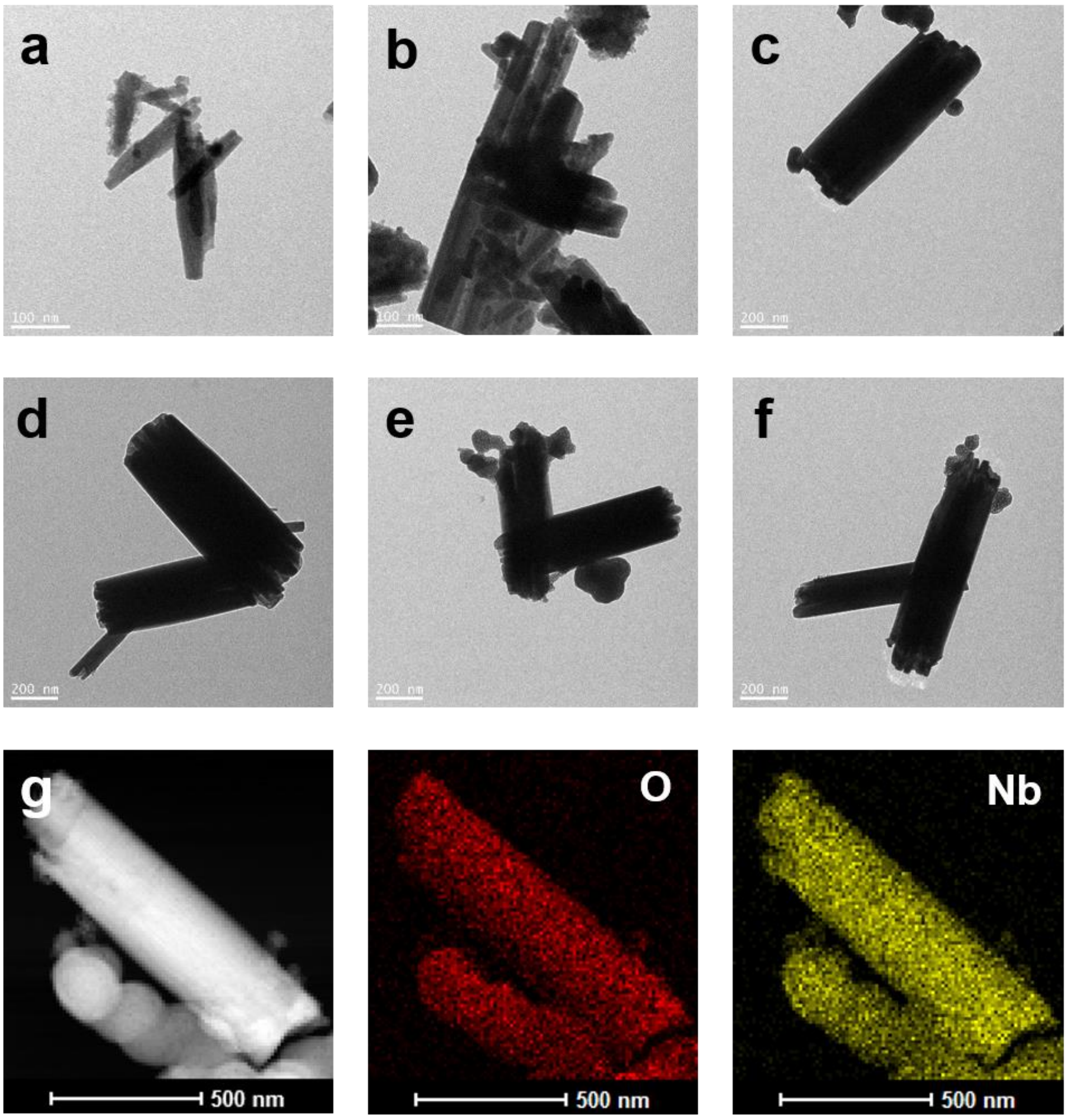

Supplementary Figure 2. TEM images of (a) T-Nb_2O_5,(b) H_2-Nb_2O_5, (c) 673K-Nb_2O_5, (d)773K-Nb_2O_5, (e) 873K-Nb_2O_5, (f)973K-Nb_2O_5, respectively. (g) STEM images of 873K-Nb_2O_5 and the elemental mapping of O and Nb

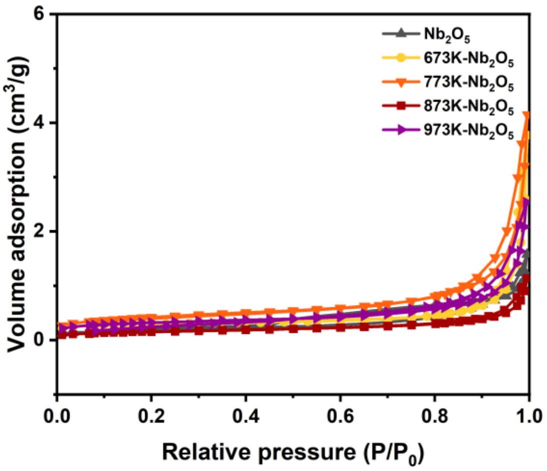

Supplementary Figure 3. Nitrogen adsorption-desorption isotherms of $NaBH_4$-treated Nb_2O_5 samples.

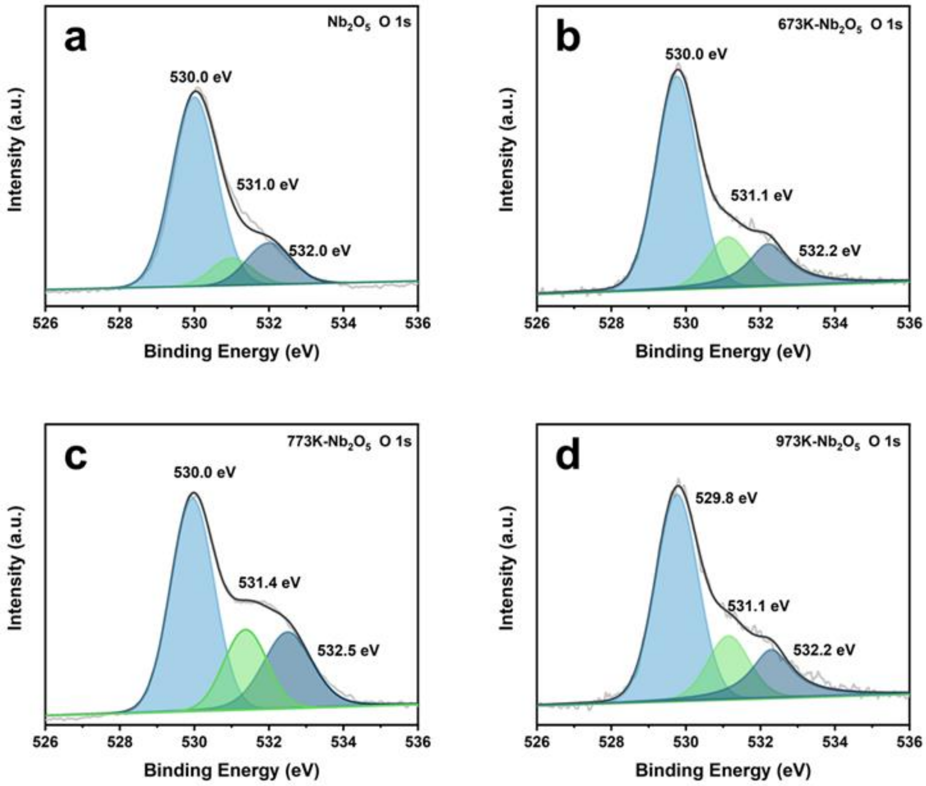

Supplementary Figure 4. O 1s XPS spectra of (a)Nb_2O_5, (b)673K- Nb_2O_5, (c)773K- Nb_2O_5, and (d)973K-Nb_2O_5.

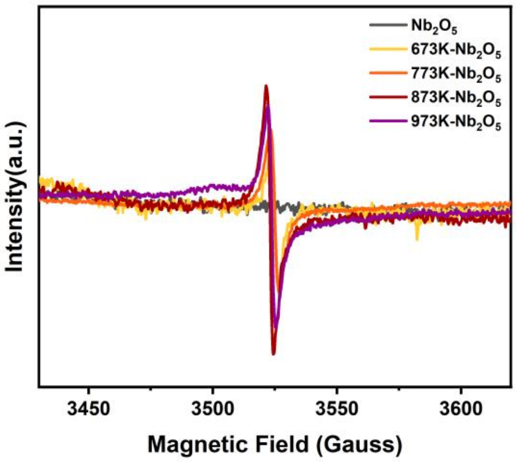

Supplementary Figure 5. EPR spectra of different $NaBH_4$-treated Nb_2O_5 samples.

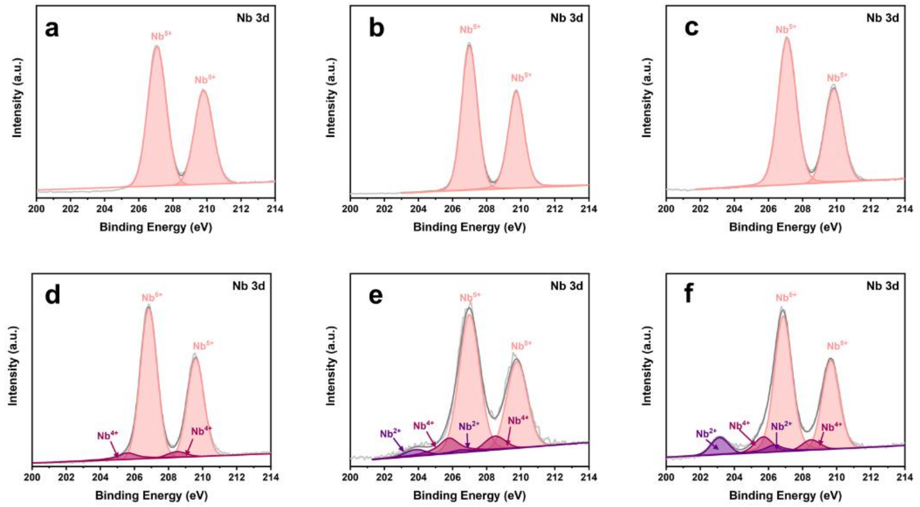

Supplementary Figure 6. Nb 3d XPS spectra of (a) Nb_2O_5, (b) H_2-Nb_2O_5, (c) 673K-Nb_2O_5, (d) 773K-Nb_2O_5, (e) 873K-Nb_2O_5 and (f) 973K-Nb_2O_5.

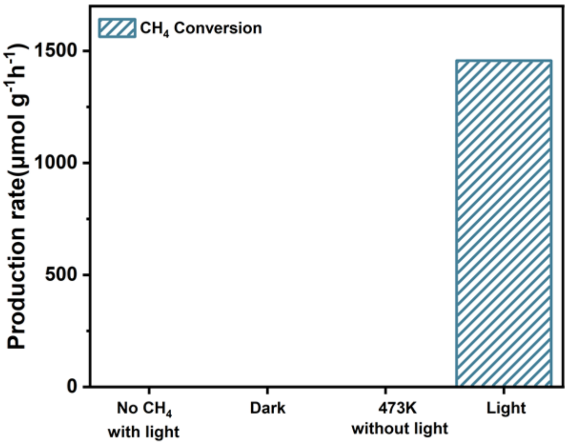

Supplementary Figure 7. Conditional comparison experiment. The detected conversion of methane with different reaction conditions, conditions from left to right: 5 mg catalyst with Xe lamp irradiation in a vacuum; 5 mg catalyst and methane without light; 5 mg catalyst and methane heating at 473K without light for 4h; 5 mg catalyst and methane with Xe lamp irradiation for 4h.

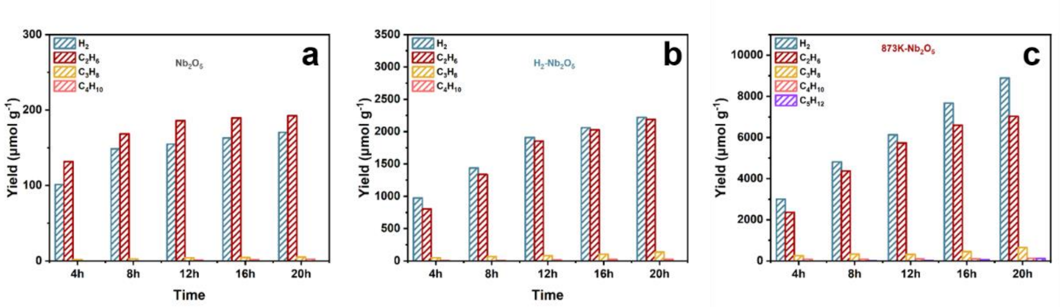

Supplementary Figure 8. The long-time photocatalytic NOCM reactions over (a)Nb_2O_5, (b) H_2-treated Nb_2O_5, and (c) 873K-Nb_2O_5.

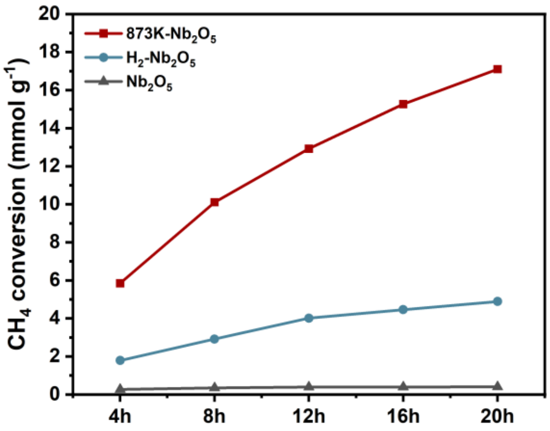

Supplementary Figure 9. Comparison of methane conversion rates for long-term reactions on samples with different treatments. Reaction condition: 5 mg catalyst, 44.6 mL methane, irradiated under 300 W Xe lamp for 20 h.

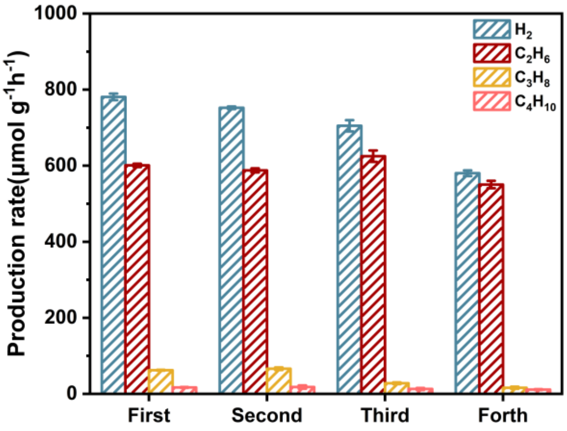

Supplementary Figure 10. The cycle photocatalytic NOCM reactions over 873K-Nb_2O_5.

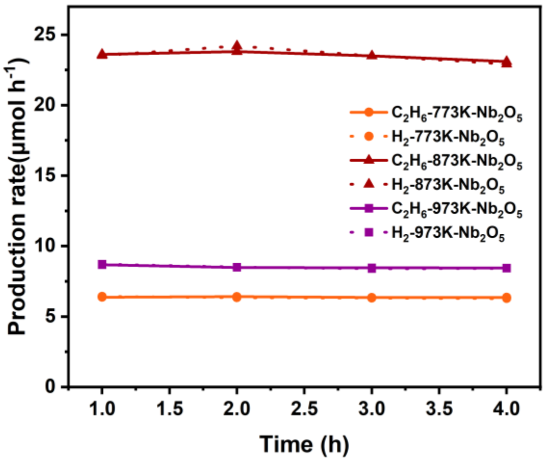

Supplementary Figure 11. Time courses of the production rates of ethane (solid line) and hydrogen (dotted line) in the flow-type photocatalytic reaction test of the NOCM over samples.

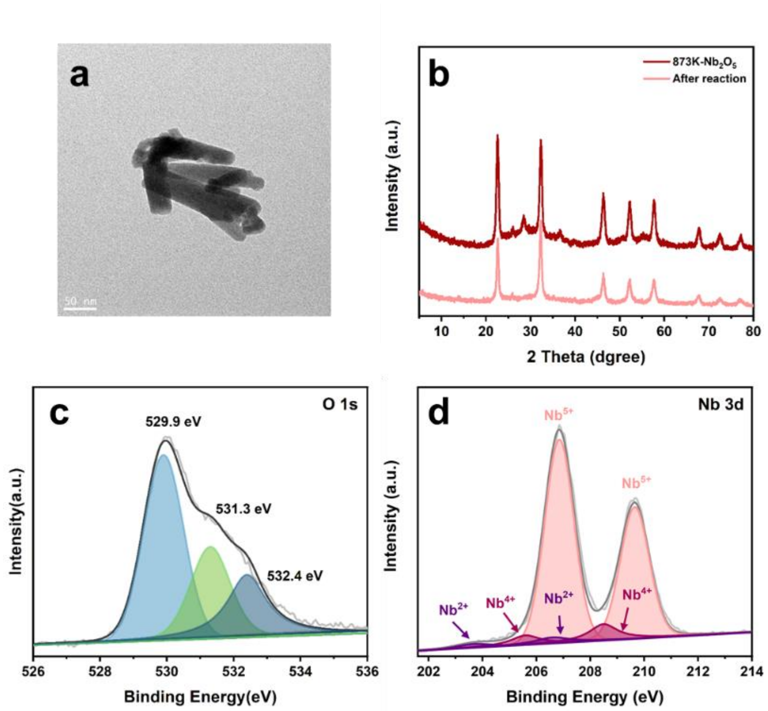

Supplementary Figure 12. The (a) TEM image, (b) XRD patterns, and (c, d) XPS analysis of 873K-Nb_2O_5 after 20h reaction.

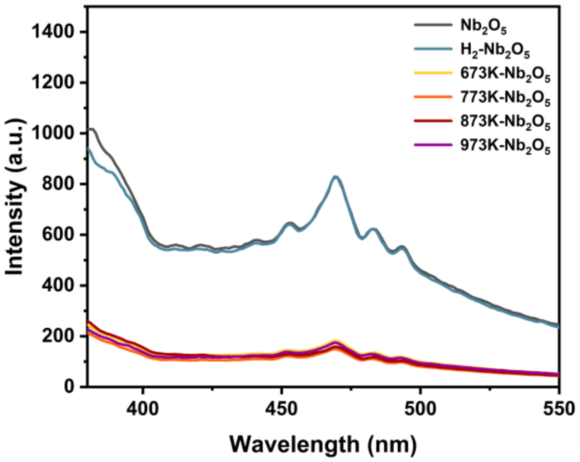

Supplementary Figure 13. Room-temperature PL emission spectra (excitation wavelength is 350 nm) of different Nb_2O_5 samples.

Supplementary Note 3:

The broad peak observed centered at 370 nm corresponds to the pristine band-edge emission of Nb_2O_5. The other complex bands obtained in range of 410~ 490 nm are assigned to the deep level emission, respectively belong to the emission of band edge free excitons, bound excitons (400–425 nm), surface defects (450–480 nm), and structural defects such as distorted NbO_6 octahedral groups (490 nm).[14, 15]

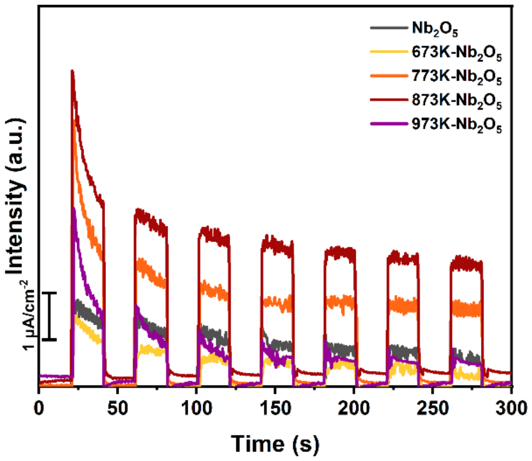

Supplementary Figure 14. Transient photocurrent responses of different Nb_2O_5 samples.

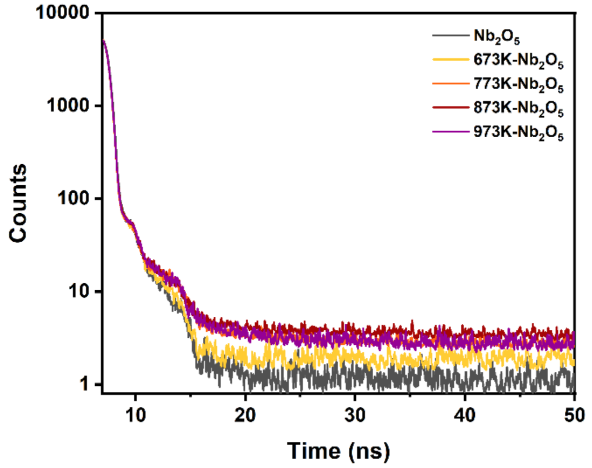

Supplementary Figure 15. The signal with an emission wavelength of 470 nm of different Nb_2O_5 samples.

Supplementary Note 4:

The values of the lifetime constants (τ) are shown in Table S10. The PL peak decay of $NaBH_4$-treated-Nb_2O_5 were slower than that of pristine and H_2-treated Nb_2O_5. The 873K-Nb_2O_5 with the longest lifetime of 3.448 ns compared to 0.066 ns of pristine Nb_2O_5, reveals a long life of photogenerated electrons in the excited state, which is highly desirable for the surface reaction. For 873K-Nb_2O_5, the double-exponential model suggested that two emissive states were involved in the PL decay with the fast decay component $τ_1$ and the much slower component $τ_2$, revealing that oxygen vacancies and various defects generated during phase transition effectively inhibit electron/hole recombination.

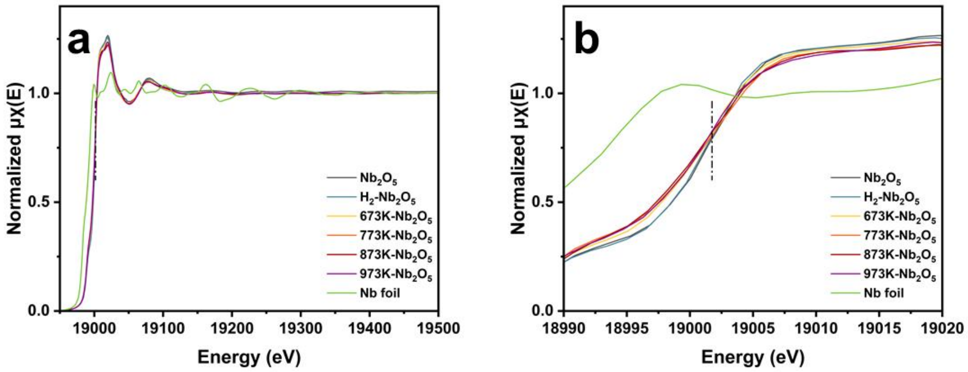

Supplementary Figure 16. (a)Normalized Nb K-edge XANES spectra of different Nb_2O_5 samples. (b)The position of the dotted line is the highest point of corresponding first derivative absorption curves of Nb_2O_5.

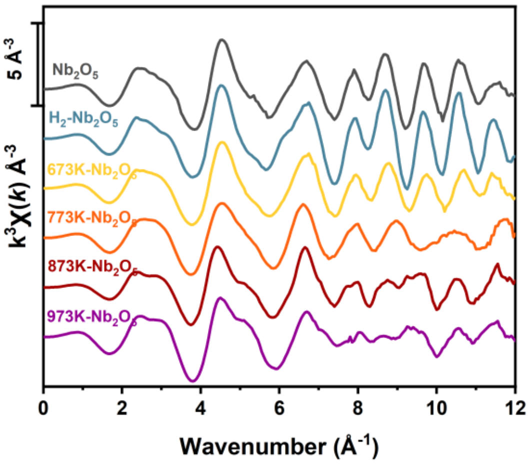

Supplementary Figure 17. Corresponding fitted k^3-weighted Nb K-edge EXAFS spectra.

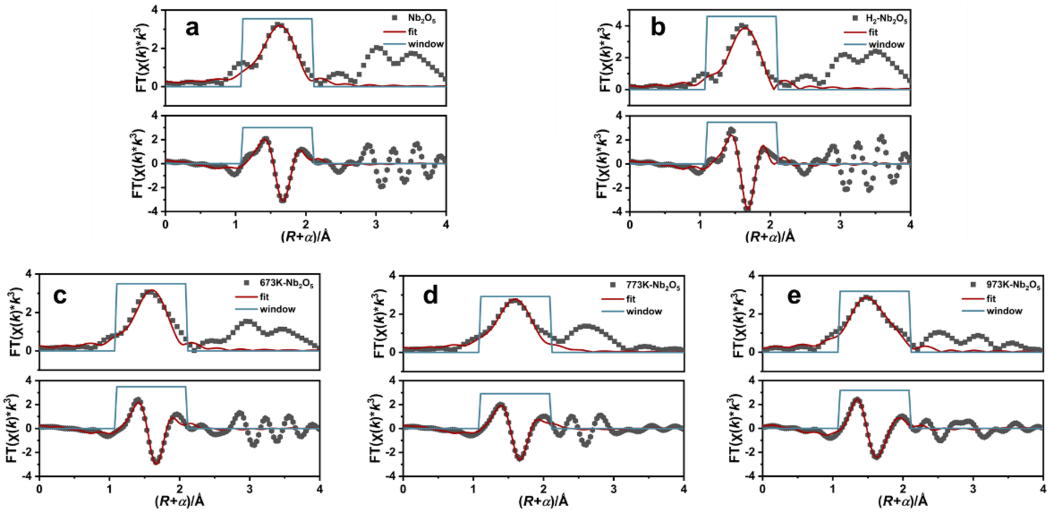

Supplementary Figure 18. Nb K-edge EXAFS (points) and curvefit (line) for (a) T-Nb_2O_5, (b) H_2-Nb_2O_5 and (c-e) $NaBH_4$-treated Nb_2O_5 in different temperature shown in k^3-weighted *R*-space (FT magnitude and imaginary component). The data are k^3-weighted and not phase-corrected.

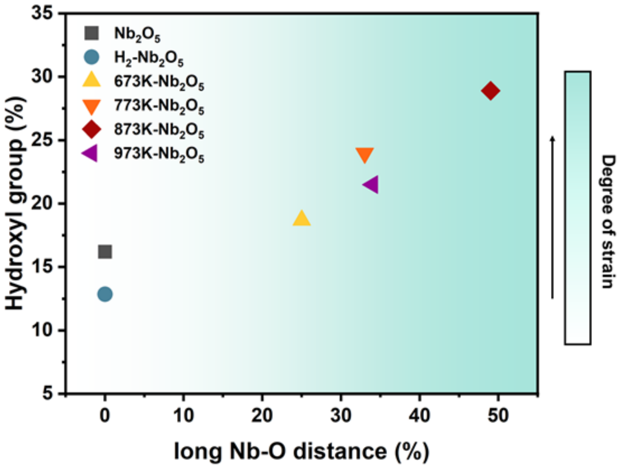

Supplementary Figure 19. The ratio of hydroxyl groups versus the ratio of longer Nb-O paths for fitting.

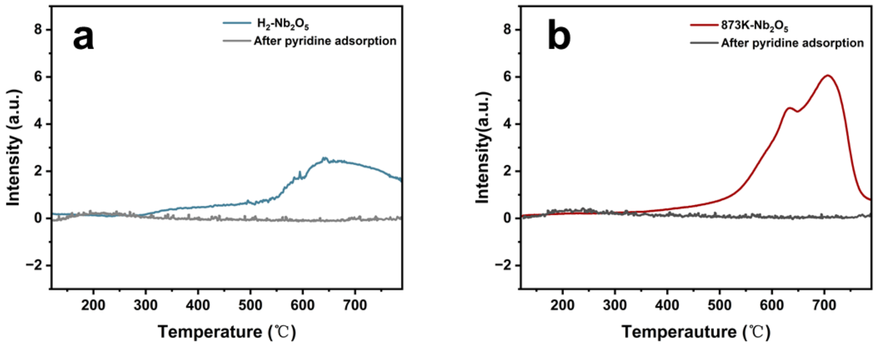

Supplementary Figure 20. NH_3-TPD spectra of (a)H_2-Nb_2O_5 and (b)873K- Nb_2O_5 before and after pyridine quenching.

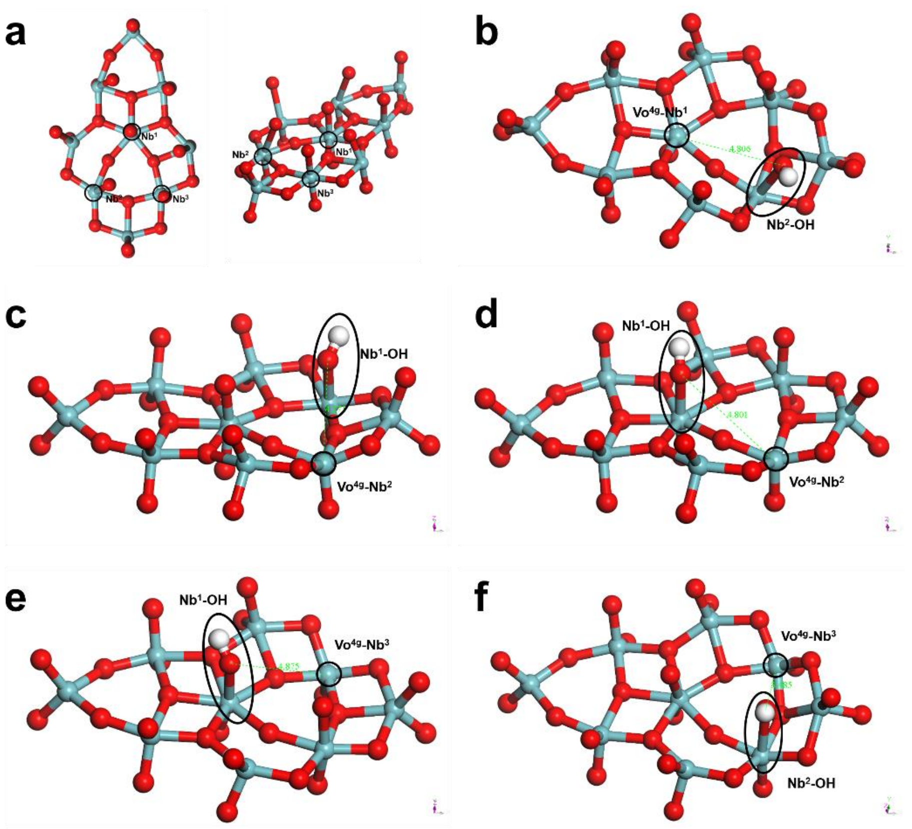

Supplementary Figure 21. (a) Structures of the T-phase Nb_2O_5 cluster from the different axis. (b-f) Structures of the dual active site FLP-Nb_2O_5 cluster (Vo^{4g}-Nb-O^{4h}-Nb-^{4g}OH). The colors of Nb, O, C, and H are blue, red, black, and white, respectively.

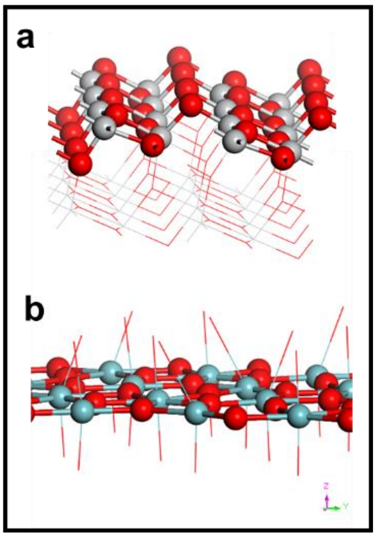

Supplementary Figure 22. X-Y plane perspective of metal-oxygen layer in (a)antase TiO_2 and (b) T-phase Nb_2O_5.

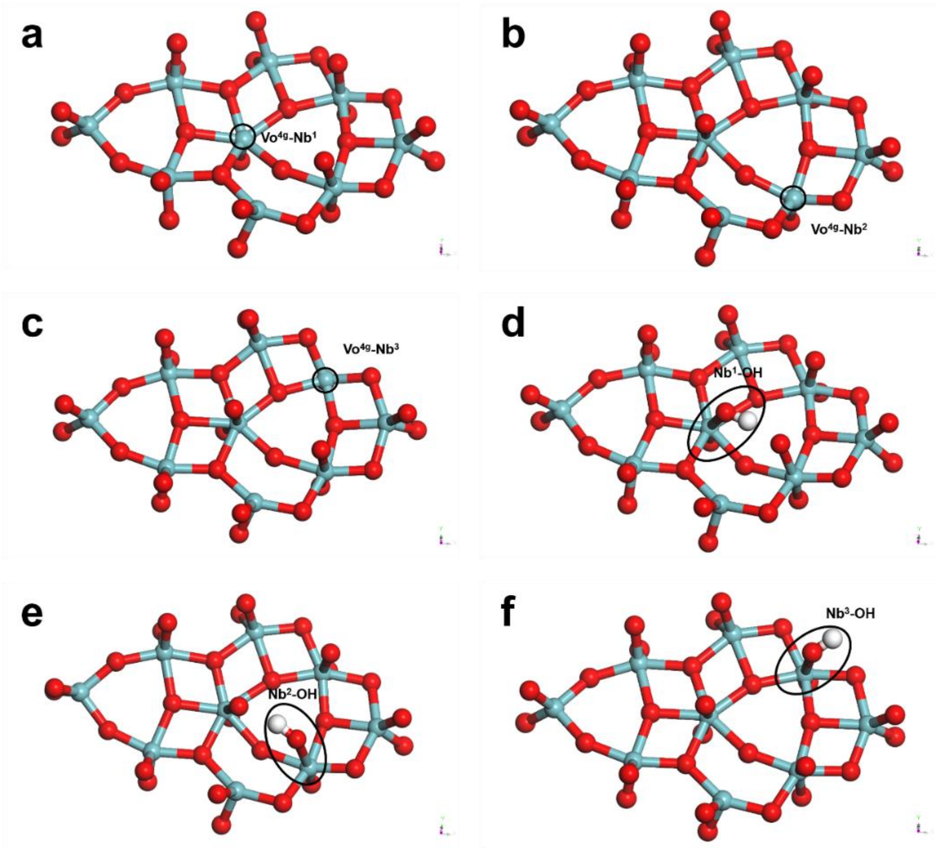

Supplementary Figure 23. (a-c) Structures of the Vo^{4g}-Nb_2O_5 cluster from the z-axis. (d-f) Structures of the Nb-^{4g}OH cluster from the z-axis. The colors of Nb, O, C, and H are blue, red, black, and white, respectively.

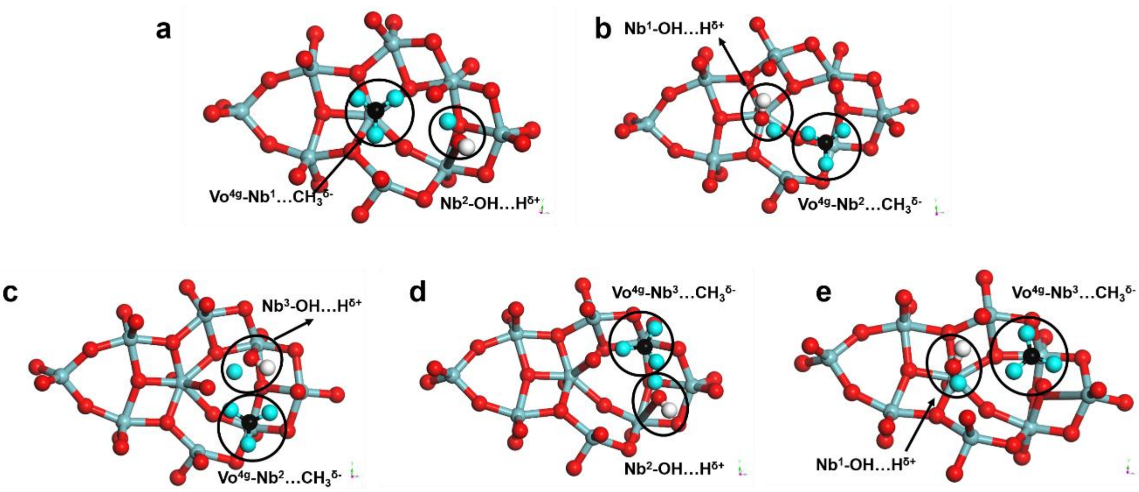

Supplementary Figure 24. (a-e) Schematic models of methane adsorption on FLP-Nb_2O_5 models. The color of Nb, O, and Care blue, red and black, respectively. The colors of H in the hydroxyl group is white, and in methane is cyan-blue.

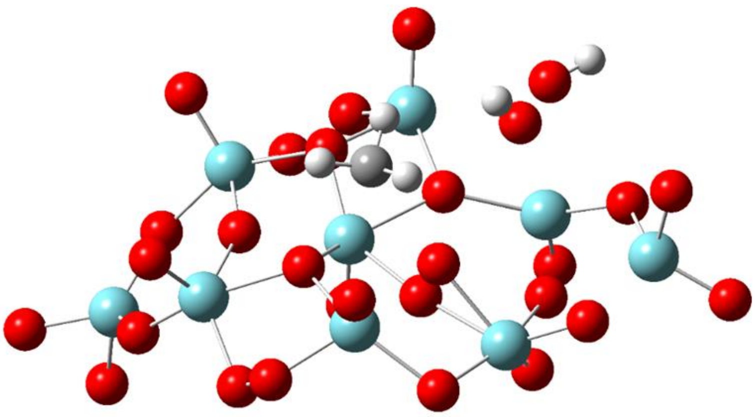

Supplementary Figure 25. The model of adsorption of $CH_3^{\delta-}$ on exposed Nb^1 site and $H^{\delta+}$ on Nb^3-OH site in ES. The green and blue groups represent the distribution of electrons and holes, respectively. The colors of Nb, O, C, and H atoms are blue, red, gray, and white, respectively.

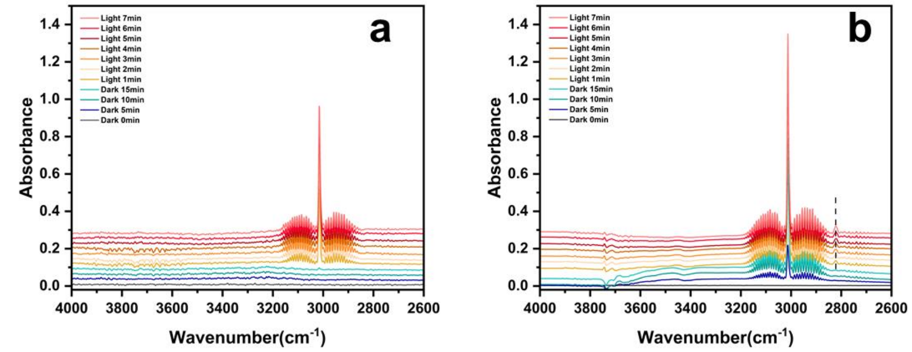

Supplementary Figure 26. The in situ diffuse reflectance infrared Fourier transform spectroscopy of (a) pristine Nb_2O_5 and (b) 873K-Nb_2O_5 under dark and light situation. (20% CH_4 gas 20 sccm, 298 K in dry condition).

Supplementary References

1.Meng L. et al. Gold plasmon-induced photocatalytic dehydrogenative coupling of methane to ethane on polar oxide surfaces. *Energy Environ. Sci.* **11**, 294-298 (2018).

2.Li N. et al. Photocatalytic coupling of methane and CO_2 into C2-hydrocarbons over Zn doped g-C_3N_4 catalysts. *Appl. Surf. Sci.* **498**, 143861 (2019).

3.Yu X. et al. Selective photocatalytic conversion of methane into carbon monoxide over zinc-heteropolyacid-titania nanocomposites. *Nat. Commun.* **10**, 700 (2019).

4.Wu S. et al. Ga-doped and Pt-loaded porous TiO_2–SiO_2 for photocatalytic nonoxidative coupling of methane. *J. Am. Chem. Soc.* **141**, 6592-6600 (2019).

5.Tahir M. Enhanced photocatalytic CO_2 reduction to fuels through bireforming of methane over structured 3D MAX Ti_3AlC_2/TiO_2 heterojunction in a monolith photoreactor. *J. CO_2 Util.* **38**, 99-112 (2020).

6.Lang J. et al. Highly efficient light-driven methane coupling under ambient conditions based on an integrated design of a photocatalytic system. *Green Chem.* **22**, 4669-4675 (2020).

7.Yu X. et al. Stoichiometric methane conversion to ethane using photochemical looping at ambient temperature. *Nat. Energy.* **5**, 511-519 (2020).

8.Chen Z. et al. Non-oxidative Coupling of Methane: N-type Doping of Niobium Single Atoms in TiO_2–SiO_2 Induces Electron Localization. *Angew. Chem. Int. Ed.* **60**, 11901-11909 (2021).

9.Jiang W. et al. Pd-Modified ZnO–Au Enabling Alkoxy Intermediates Formation and Dehydrogenation for Photocatalytic Conversion of Methane to Ethylene. *J. Am. Chem. Soc.* **143**, 269-278 (2021).

10.Ma J. et al. Exploring the Size Effect of Pt Nanoparticles on the Photocatalytic Nonoxidative Coupling of Methane. *ACS Catal.* **11**, 3352-3360 (2021).

11.Wang G. et al. Light‐Induced Nonoxidative Coupling of Methane Using Stable Solid Solutions. *Angew. Chem. Int. Ed.* **133**, 20928-20932 (2021).

12.Li X. et al. Platinum- and CuO_x-Decorated TiO_2 Photocatalyst for Oxidative Coupling of Methane to C2 Hydrocarbons in a Flow Reactor. *Angew. Chem. Int. Ed.* **59**, 19702-19707 (2020).

13.G. Herzberg, Molecular Spectra and Molecular Structure (Krieger, Malabar, FL, 1989)

14.Jia Y. et al. Theoretical and Experimental Study on Exciton Properties of TT-, T-, and H-Nb_2O_5. *J. Phys. Chem. C.* **124**, 15066-15075 (2020).

15. Usha N. et al. Mixed Nb_2O_5: MoO_3 (95: 5 and 85: 15) thin films and their properties for electrochromic device applications. *J. Mater. Sci. Mater. Electron.* **27**, 7809-7821 (2016).
